# Successful lung-protective ventilatory management during the VV-ECMO in a severe COVID-19 pneumonia patient with extensive pneumomediastinum and subcutaneous emphysema: a case report

**DOI:** 10.1186/s40981-022-00505-8

**Published:** 2022-02-21

**Authors:** Jumpei Kohara, Shinichi Kai, Kazuya Hashimoto, Yudai Takatani, Naoya Tanabe, Satoshi Hamada, Kosai Cho, Tomoharu Tanaka, Isao Ito, Shigeru Ohtsuru

**Affiliations:** 1 Department of Anesthesia, Kyoto University Hospital, Kyoto, Japan; 2 Department of Primary Care and Emergency Medicine, Kyoto University Graduate School of Medicine, Kyoto, Japan; 3 Department of Respiratory Medicine, Graduate School of Medicine, Kyoto University, Kyoto, Japan; 4 Department of Advanced Medicine for Respiratory Failure, Graduate School of Medicine, Kyoto University, Kyoto, Japan

**Keywords:** COVID-19, Pneumomediastinum/subcutaneous emphysema, Veno-venous extracorporeal membrane oxygenation, Lung-protective ventilation

## Abstract

**Background:**

Ventilatory management of respiratory failure with pneumomediastinum/subcutaneous emphysema is not established. Herein, we report a case of severe COVID-19 pneumonia with extensive pneumomediastinum/subcutaneous emphysema, rescued by thorough lung-protective ventilatory management after applying the VV-ECMO.

**Case presentation:**

A 68-year-old male with no medical history was admitted to a local hospital and diagnosed with COVID-19 pneumonia. His pulmonary parameters worsened during invasive ventilation due to the development of pneumomediastinum/subcutaneous emphysema, and then he was transferred to our hospital. On arrival, we immediately decided to apply VV-ECMO and switch to ultraprotective ventilation. After maintaining the initial ventilation with a neuromuscular blocking agent for 2 days, we gradually increased PEEP while limiting PIP to 25 cmH_2_O. The patient was weaned off VV-ECMO on day 10; he was transferred to the medical ward after extubation.

**Conclusions:**

Lung-protective ventilatory management should be performed thoroughly during VV-ECMO in severe COVID-19 pneumonia with pneumomediastinum/subcutaneous emphysema.

## Background

Pneumomediastinum and subcutaneous emphysema have been noted to complicate COVID-19 [1]. These complications make it difficult to maintain adequate gas exchange by conventional mechanical ventilation and prevent further ventilator-induced lung injury (VILI). The use of veno-venous extracorporeal membrane oxygenation (VV-ECMO) to switch to ultraprotective ventilation has been reported as a helpful tool [2]. However, ventilatory management of respiratory failure with pneumomediastinum/subcutaneous emphysema during VV-ECMO is not established. Herein, we report a severe COVID-19 pneumonia patient with extensive pneumomediastinum/subcutaneous emphysema rescued by thorough lung-protective ventilation management after applying the VV-ECMO.

## Case presentation

A 68-year-old male (height 178 cm, weight 89 kg, BMI 28 kg/m^2^) with no significant past medical history was admitted to a local hospital for fever and diagnosed with COVID-19 pneumonia. Although favipiravir and dexamethasone were administered for COVID-19, his respiratory function progressively deteriorated, requiring endotracheal intubation and invasive ventilation. The mechanical ventilator setting was pressure control (PC) mode with FiO_2_ 0.65, positive end-expiratory pressure (PEEP) 15 cmH_2_O, peak inspiratory pressure (PIP) 30 cmH_2_O, and respiratory rate (RR) 15/min (PaO_2_ 96.0 mmHg and PaCO_2_ 48.4 mmHg). On the 6th day of post-hospitalization, his pulmonary parameters worsened due to the development of pneumomediastinum/subcutaneous emphysema, and he was transferred to our hospital.

**Fig. 1:**
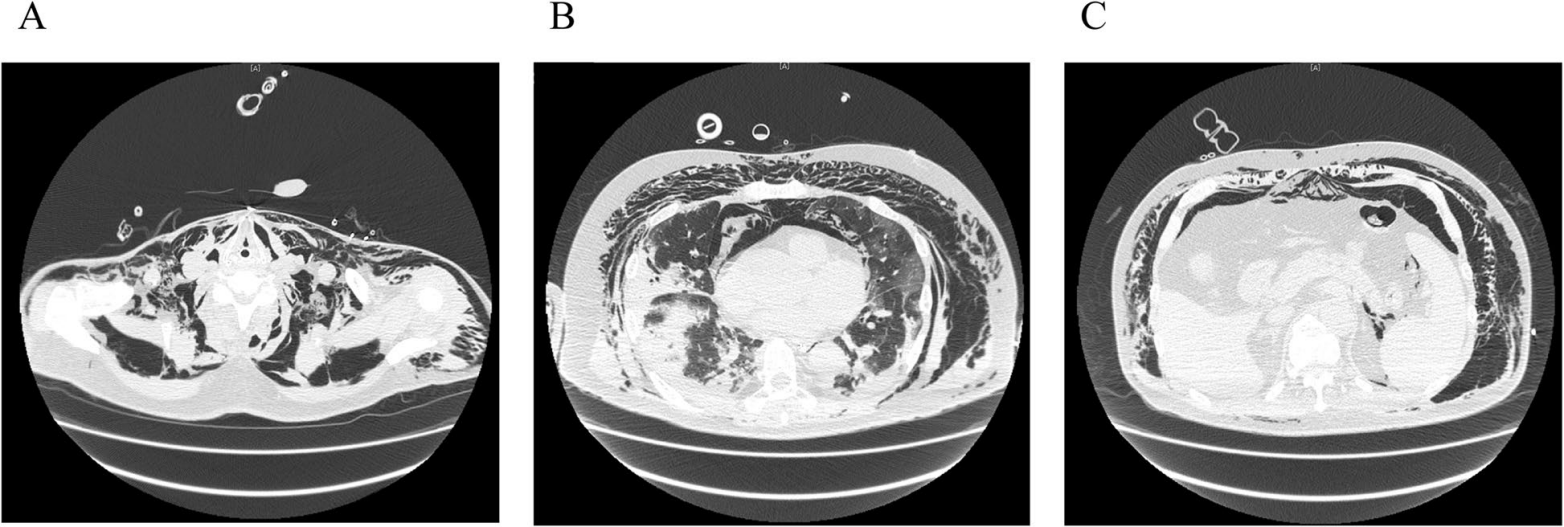
Chest CT on admission. **A** At the level of the neck. **B** Lung bases. **C** At the level of the pancreas. These images show bilateral diffuse ground-glass opacity with extensive basal consolidation and extensive pneumomediastinum/subcutaneous emphysema from the neck to abdomen

**Fig. 2:**
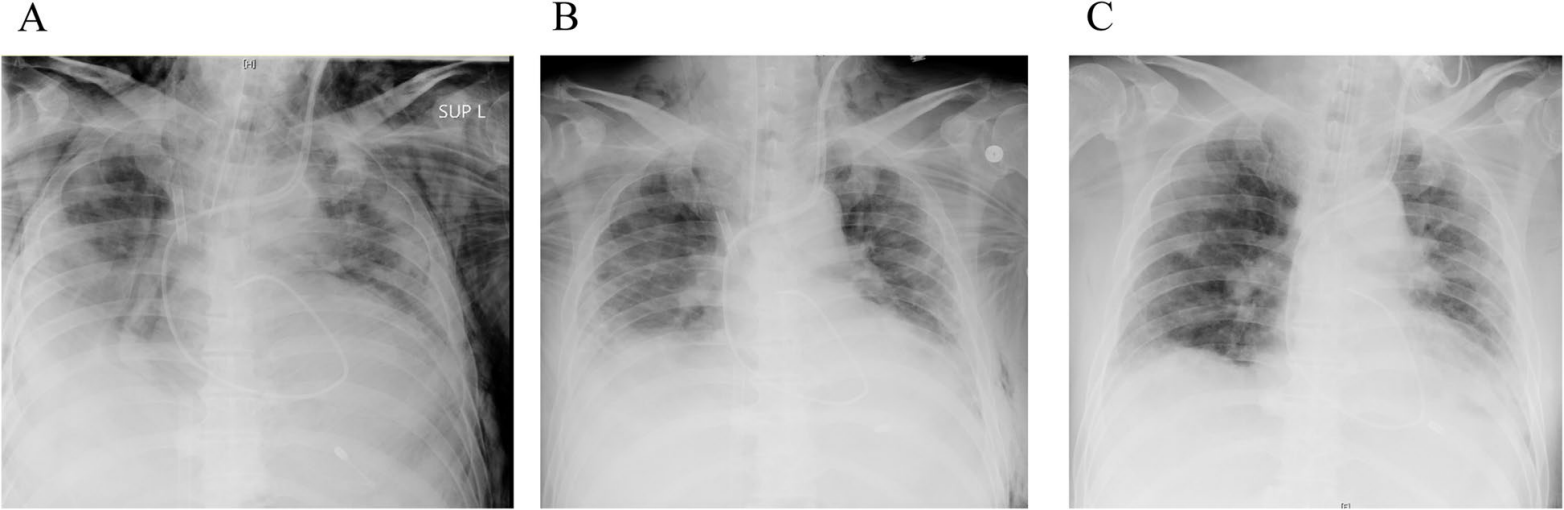
Chest X-ray of the patient. **A** On admission. **B** Fourth day post-ECMO cannulation. **C** Post-ECMO decannulation. These X-rays showed that extensive pneumomediastinum/subcutaneous emphysema gradually decreased

PaO_2_/FiO_2_ was 92 mmHg on 0.75 of FiO_2_ and 10 cmH_2_O PEEP. The patient was also in respiratory acidosis (PaCO_2_ 70.6 mmHg and pH 7.293), and his Murray score was 3.5 (Table 1). The crepitus was noted on palpation throughout his body (from the neck to the knee). Chest X-ray showed subcutaneous emphysema with pneumomediastinum in addition to bilateral pulmonary infiltrates (Fig. 1A). A CT scan also revealed bilateral diffuse ground-glass opacity with extensive basal consolidation and extensive pneumomediastinum/subcutaneous emphysema (Fig. 2). His laboratory workup was as follows: WBC 11,570/mm^3^, Hb 15.4 g/dL, platelets 17.5 × 10^4^/mm^3^, C-reactive protein 19.4 mg/dL, and d-dimer 5.7 μg/mL. His renal function, liver function, and coagulation profile were within normal limits. We administered remdesivir, instead of favipiravir, together with dexamethasone.

**Table 1:** Ventilator parameters and arterial blood gas analysis

	Pre-ECMO	ECMO day 1	ECMO day 5	ECMO day7	ECMO weaning	Post-ECMO	Pre-extubation
Mode	SIMV	PCV	PCV	PCV	PCV	PCV	CPAP/PS
FiO_2_	0.75	0.4	0.4	0.4	0.5	0.5	0.5
PIP/PS	25	10	18	22	24	25	5
PEEP	10	5	10	12	12	10	5
RR	22	10	10	8	16	20	–
TV	–	10	180	280	395	545	–
SG FiO_2_	–	0.9	0.9	0.9	0.21	–	–
SG flow	–	3	3.5	3.5	1.0	–	–
EBF	–	3.9	3.9	3.9	1.4	–	–
pH	7.29	7.36	7.42	7.41	7.39	7.46	7.53
PaO_2_	69	56.5	78.0	77.0	102.3	93.5	155.4
PaCO_2_	70.6	53.8	46.4	44.8	51.3	39.5	34.3

*SIMV* Synchronized intermittent mandatory ventilation, *PCV* Pressure control ventilation, *CPAP/PS* Continuous positive airway pressure/pressure support, *FiO*_*2*_ Fraction of inspired oxygen, *PIP* Peak inspiratory pressure (cmH_2_O), *PEEP* Positive end-expiratory pressure (cmH_2_O), *RR* Respiratory rate (/min), *TV* Tidal volume (mL), *SG flow* Sweep gas flow (L/min), *EBF* Extracorporeal blood flow (3.9 L/min), *PaO*_*2*_ Partial pressure of oxygen in arterial blood (mmHg), *PaCO*_*2*_ Partial pressure of carbon dioxide in arterial blood (mmHg)

VV-ECMO was immediately applied because we could not maintain optimal gas exchange by conventional ventilation while preventing VILI. A 25-French drainage cannula was inserted percutaneously into the inferior vena cava via left femoral approach, and a 23-French return cannula was inserted into the inferior vena cava via right femoral approach under fluoroscopy guidance. After VV-ECMO application, the pump flow was adjusted to a target percutaneous oxygen saturation of 95–100%. The sweep gas flow was titrated to a target arterial carbon dioxide partial pressure of 45–50 cmH_2_O (blood flow 3.9 L/min, sweep gas flow 3 L/min, and FiO_2_ 0.9), and heparin anticoagulation was adjusted to maintain an activated partial thrombin time of 50–60 s. The mechanical ventilation setting was switched to ultraprotective ventilation using pressure control ventilation (PCV) with PIP 10 cmH_2_O, PEEP 5 cmH_2_O, RR 10/min, and FiO_2_ 0.4 (Table 1). We held the initial ventilation setting with a continuous neuromuscular blocking agent for 2 days. On the 5th day after ECMO initiation, we increased PEEP gradually up through 12 cmH_2_O while limiting PIP to 25 cmH_2_O. We also performed clinical and radiological assessments daily to check for exacerbation of pneumomediastinum/subcutaneous emphysema (Fig. 1B, C). ECMO weaning was started on the 9th day after ECMO initiation because chest X-ray findings and the patient’s respiratory parameters progressively improved. The extracorporeal blood flow was reduced to 1.4 L/min, and the sweep gas flow was reduced to 1 L/min (FiO_2_ 0.21), while the ventilator was set at PC mode with FiO_2_ 0.5, PEEP 12 cmH_2_O, PIP 24 cmH_2_O, and RR 16/min. We confirmed if the patient could maintain oxygenation and ventilation for 24 h. Although we finally turned off the sweep gas flow, the patient’s oxygenation and ventilation were adequate. Then, decannulation was performed on the 10th day after ECMO initiation. Although some blood clots were detected in the ECMO circuit, there were no complications related to ECMO support. Although his respiratory status temporarily worsened with fever, we performed antibiotic therapy, extubating on day 29 (Table 1). CT on day 33 showed decreased basal consolidation with a slight peripheral ground-glass opacity and no pneumomediastinum/subcutaneous emphysema. He was transferred to the medical ward because of his respiratory improvement.

## Discussion

We present a case of severe COVID-19 pneumonia with extensive pneumomediastinum/subcutaneous emphysema rescued by thorough lung-protective ventilatory management after applying the VV-ECMO. The ultraprotective ventilation might induce alveolar derecruitment when PEEP is not adequately titrated. Therefore, we increased PEEP gradually to open collapsed alveoli on the 5th day after ECMO initiation while limiting PIP to 25 cmH_2_O. Thorough lung-protective ventilatory management during VV-ECMO might be essential to protect the lungs from VILI.

The pneumomediastinum/subcutaneous emphysema frequently occurs in COVID-19 patients, which is a risk factor for higher mortality [1]. Although pneumomediastinum/subcutaneous emphysema has traditionally been related to the development of high airway pressure associated with high tidal volume ventilation, pneumomediastinum/subcutaneous emphysema in COVID-19 infection does not appear to be associated with the traditional barotrauma mechanism. Lemmers et al. found that COVID-19 patients with ARDS developed pneumomediastinum/subcutaneous emphysema despite applying lung-protective ventilation [3]. It might be possible that undiagnosed tracheal damage in COVID-19 patients can cause pneumomediastinum/subcutaneous emphysema in the absence of extremely high airway pressure. Fiacchini et al. reported that tracheal damage occurs in almost half of the ventilated patients with COVID-19. The high rate of tracheal damage is associated with pronation maneuvers, higher doses of steroids, and a lower PaO_2_/FiO_2_ ratio [4]. Therefore, an adequate clinical and radiological assessment should be performed in ventilated patients with COVID-19 even if protective mechanical ventilation was applied.

The management of mechanical ventilation in patients with severe COVID-19 pneumonia and pneumomediastinum/subcutaneous emphysema is challenging because we need to maintain a balance between providing adequate gas exchange and preventing worsening of pneumomediastinum/subcutaneous emphysema. The Extracorporeal Life Support Organization guidelines list carbon dioxide retention on mechanical ventilation despite high plateau pressures (> 30 cmH_2_O) and severe air leak syndrome as indications for initiating extracorporeal life support [5]. Therefore, we immediately initiated VV-ECMO and switched to ultraprotective ventilation. Some studies have shown that VV-ECMO significantly improves survival in severe acute respiratory failure [6] and air leak syndrome, such as pneumothorax, pneumomediastinum, and subcutaneous emphysema [2]. Although the timing for initiation of V-V ECMO in severe respiratory failure remains debatable, we decided to start VV-ECMO promptly because a previous study observed that increased pre-ECMO ventilation duration is associated with worse outcomes [7]. Furthermore, early initiation of ECMO for lung protection might be a suitable treatment to avoid unnecessary surgical procedures and aerosol generation [8]. Subcutaneous drains and intrapleural chest drains should be inserted in worsening pneumomediastinum, with associated mediastinal shift and respiratory compromise [9].

After introducing VV-ECMO, the initial PEEP was set as 5 cmH_2_O because pulmonary barotrauma from mechanical ventilation, especially with high PEEP but not plateau pressure, is a well-known risk factor for pneumomediastinum [10]. We also used a neuromuscular drug for 2 days to avoid the occurrence of coughing spells. On the other hand, insufficient PEEP level might lead to progressive pulmonary derecruitment during ultraprotective ventilation with low tidal volume [11]. Therefore, we increased PEEP gradually up through 12 cmH_2_O while checking for exacerbation of pneumomediastinum/subcutaneous emphysema. In some clinical studies, PEEP also was set more than 10 cmH_2_O in ECMO-supported patients with ARDS [5]. However, the optimal PEEP level has been challenging to determine because it varies widely among ARDS patients during ultraprotective ventilation. In such a case, electrical impedance tomography might be a helpful tool for real-time monitoring of the PEEP effect [12].

Terragni et al. found that the values of plateau pressure lower than 28 cmH_2_O were associated with less tidal hyperinflation than values of plateau pressure ranging between 28 and 30 cmH_2_O in the ventilated patients with ARDS [13]. We also limited PIP to 25 cmH_2_O in order to prevent exacerbation of pneumomediastinum/subcutaneous emphysema throughout the ECMO period. Limiting tidal volume to 6 mL/kg predicted body weight and plateau pressure to 30 cmH_2_O, which the ARDS Network recommends, might be insufficient in patients characterized by a larger amount of collapsed lung [13]. Even after applying VV-ECMO, thorough lung-protective ventilatory management might be essential to allow the lungs to rest, reduce lung inflation, and avoid overdistension.

## Conclusion

In conclusion, we experienced a severe COVID-19 pneumonia patient with extensive pneumomediastinum/subcutaneous emphysema, rescued by thorough ventilatory management after applying VV-ECMO. After the initial ventilation was maintained for 2 days, we gradually increased PEEP while limiting PIP to 25 cmH_2_O. Thorough lung-protective ventilatory management during VV-ECMO might be essential to protect the lungs from VILI.

## Data Availability

Not applicable.

## Abbreviations

VV-ECMO: Veno-venous extracorporeal membrane oxygenation
VILI: Ventilator-induced lung injury
ARDS: Acute respiratory distress syndrome
PEEP: Positive end-expiratory end pressure
PIP: Peak inspiratory pressure
RR: Respiratory rate
PCV: Pressure control ventilation
